# Use of noninvasive ventilation in immunocompromised patients with acute respiratory failure: a systematic review and meta-analysis

**DOI:** 10.1186/s13054-016-1586-9

**Published:** 2017-01-07

**Authors:** Hui-Bin Huang, Biao Xu, Guang-Yun Liu, Jian-Dong Lin, Bin Du

**Affiliations:** 1 Medical ICU, Peking Union Medical College Hospital, Peking Union Medical College and Chinese Academy of Medical Sciences, 1 Shuai Fu Yuan, Beijing, 100730 People’s Republic of China; 2 Department of Critical Care Medicine, the First Affiliated Hospital of Fujian Medical University, 20 Chazhong Road, Fuzhou, 350000 People’s Republic of China; 3 Critical Care Medicine Center, the PLA 302 Hospital, No. 100 Xisihuanzhong Road, Fengtai District, Beijing, 100039 People’s Republic of China

**Keywords:** Acute respiratory failure, Noninvasive ventilation, Immunocompromised patients, Meta-analysis

## Abstract

**Background:**

Acute respiratory failure (ARF) remains a common hazardous complication in immunocompromised patients and is associated with increased mortality rates when endotracheal intubation is needed. We aimed to evaluate the effect of early noninvasive ventilation (NIV) compared with oxygen therapy alone in this patient population.

**Methods:**

We searched for relevant studies in MEDLINE, EMBASE, and the Cochrane database up to 25 July 2016. Randomized controlled trials (RCTs) were included if they reported data on any of the predefined outcomes in immunocompromised patients managed with NIV or oxygen therapy alone. Results were expressed as risk ratio (RR) and mean difference (MD) with accompanying 95% confidence interval (CI).

**Results:**

Five RCTs with 592 patients were included. Early NIV significantly reduced short-term mortality (RR 0.62, 95% CI 0.40 to 0.97, *p* = 0.04) and intubation rate (RR 0.52, 95% CI 0.32 to 0.85, *p* = 0.01) when compared with oxygen therapy alone, with significant heterogeneity in these two outcomes between the pooled studies. In addition, early NIV was associated with a shorter length of ICU stay (MD −1.71 days, 95% CI −2.98 to 1.44, *p* = 0.008) but not long-term mortality (RR 0.92, 95% CI 0.74 to 1.15, *p* = 0.46).

**Conclusions:**

The limited evidence indicates that early use of NIV could reduce short-term mortality in selected immunocompromised patients with ARF. Further studies are needed to identify in which selected patients NIV could be more beneficial, before wider application of this ventilator strategy.

**Electronic supplementary material:**

The online version of this article (doi:10.1186/s13054-016-1586-9) contains supplementary material, which is available to authorized users.

## Background

Over the past decades, immunocompromised patients have become more and more popular, due to advances in chemotherapy and bone marrow and organ transplantation [1, 2]. However, these patients are at high risk of a number of life-threatening complications, especially acute respiratory failure (ARF) [3, 4]. A variety of disease processes may induce ARF, such as immunocompromised-related opportunistic infections, pulmonary damage secondary to malignancy, drug-related pulmonary toxicity, or unidentified causes [5]. Once these patients develop ARF, they often require intensive care unit (ICU) admission and intubation for mechanical ventilation. Nevertheless, invasive mechanical ventilation (IMV) in this situation is associated with a significantly increased mortality rate ranging from 40% to 90% [3, 6]. Therefore, noninvasive ventilation (NIV), which is administrated without the use of an endotracheal tube, has increasingly attracted attention.

Theoretically NIV can be used as an alternative to IMV in treating immunocompromised patients. The beneficial effects of NIV in ARF include lung recruitment with proper use of PEEP, improvement in hypoxia and dyspnea, and relief of respiratory muscle fatigue [7]. In addition, applying NIV in immunocompromised patients can avoid side effects directly related to endotracheal intubation and IMV, such as ventilator-associated pneumonia, excessive sedation, upper-airway injuries and tracheomalacia, thus it can lead to a better clinical outcome [8].

Although a large number of case series and observational studies suggest that NIV could reduce the rate of intubation and thus the associated infections in these patients [9], the number of randomized controlled trials (RCTs) is still very limited. The 2011 Canadian guidelines made a weak recommendation (grade 2B) favoring the use of NIV in immunocompromised patients with ARF [1]. However, this recommendation remains controversial as it is based on only two small early RCTs [10, 11]. Furthermore, the results of several RCTs have been reported in recent years, and some of these trials have a modest sample size, while the conclusions are inconsistent [12–14].

Therefore, we aimed to perform a systematic review and meta-analysis of all available RCTs comparing early use of NIV with oxygen therapy alone in immunocompromised patients, to determine if differences exist between these two strategies in terms of overall mortality, rate of intubation and length of ICU stay.

## Methods

### Search strategy and selection criteria

We searched RCTs in MEDLINE, EMBASE, and the Cochrane database from inception through 25 July 2016 to identify potentially relevant studies. Search terms included: “non-invasive ventilation,” “noninvasive ventilation,” “NIV,” “continuous positive airway pressure,” “noninvasive mechanical ventilation,” “NIMV,” “BiPAP,” “CPAP,” “noninvasive positive-pressure ventilation,” “NPPV,” “hematologic,” “hematological,” “transplant,” “tumor,” “cancer,” “immunosuppression,” “immunosuppressed,” and “immunocompromised”. Our research was limited to RCTs with no language restriction. Reference lists of relative articles were also reviewed. Studies were included if they met the following criteria: (1) study design: RCT; (2) study population: immunocompromised adult patients with ARF; (3) intervention: early use of NIV compared to oxygen therapy alone; and (4) predefined outcomes: mortality, intubation rate, and length of ICU stay. We excluded studies of patients younger than age 18 years, and publications available only in abstract form or as meeting reports. We contacted the authors if the data on predefined outcomes from their studies were required.

### Data extraction and quality assessment

Two reviewers (H-BH and BX) independently extracted data from the included studies, such as the name of first author, year of publication, country, sample size, study design, setting, treatment algorithms in the study and control groups, severity of illness, and all predefined outcomes.

The aforementioned independent reviewers (H-BH and BX) evaluated the quality of studies using the risk of bias tool recommended by the Cochrane Collaboration [15]. We assigned a value of high, unclear, or low to the following items: sequence generation; allocation concealment; blinding; incomplete outcome data; selective outcome reporting; and other sources of bias. As blinding of caregivers, patients, and family members was impossible in these trials, we considered blinding only at the data collection level. Discrepancies were identified and resolved through discussion.

### Outcomes and statistical analysis

The primary outcome was short-term mortality. We defined short-term mortality as ICU, hospital, or 28-day mortality. If a study reported all of these outcome measures, the longest observation period was preferred. Secondary outcomes included intubation rate, length of ICU stay (defined as the time from admission to ICU discharge) and long-term mortality defined as mortality occurring after 3 months of follow-up. Testing the robustness of our primary outcome and exploring the influence factors of mortality, we conducted further analyses by pooled studies with the following: (1) ICU mortality; (2) hospital mortality; (3) 28-day mortality; (4) ratio of arterial pressure of oxygen/fraction of inspired oxygen (PaO_2_/FiO_2_); and (5) underlying disease.

The results from all relevant studies were combined to estimate the pooled risk ratio (RR) and associated 95% confidence intervals (CIs) for dichotomous outcomes. As to the continuous outcomes, the mean difference (MD) and 95% CI was estimated as the effect results. Some studies reported the median as the measure of treatment effect, with accompanying interquartile range (IQR). Before data analysis, we estimated the mean from the median and standard deviation (SD) from the IQR using the methods described in previous studies [16]. Heterogeneity was tested by using the *I* ^2^ statistic. An *I* ^2^ < 50% was considered to indicate insignificant heterogeneity and a fixed-effect model was used, whereas a random-effect model was used in cases of significant heterogeneity (*I* ^2^ > 50%). Sensitivity analyses were performed by excluding trials that potentially biased the results. In addition, we conducted statistical analyses when data from at least two RCTs were available. Publication bias was evaluated by visually inspecting funnel plots when at least 10 studies were included in this meta-analysis. A *p* value less than 0.05 was considered statistically significant. All statistical analyses were performed using STAT Version 12.0 and Review Manager, Version 5.3.

## Result

### Study selection

**Fig 1:**
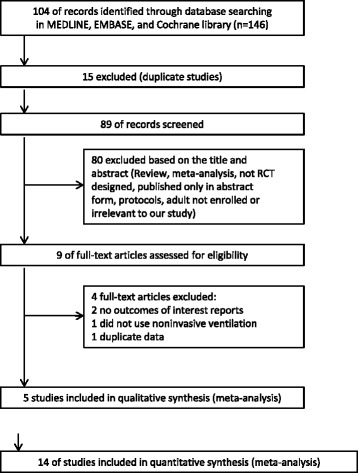
Selection process for randomized controlled trails (*RCTs*) included in the meta-analysis

A flowchart of the search strategy and the reasons for exclusion are shown in Fig. 1. The initial search yielded 104 potentially relevant studies. There were 15 studies excluded because of duplicates, and 80 studies were excluded based on reviews of the title and abstract. Thus, 9 full-text studies were read for further evaluation, and of these, 4 were excluded because they did not report predefined outcomes or meet our inclusion criteria. Finally, the remaining 5 RCTs, which enrolled 592 patients, were included in our analysis [10–14].

### Study characteristics and quality

**Fig 2:**
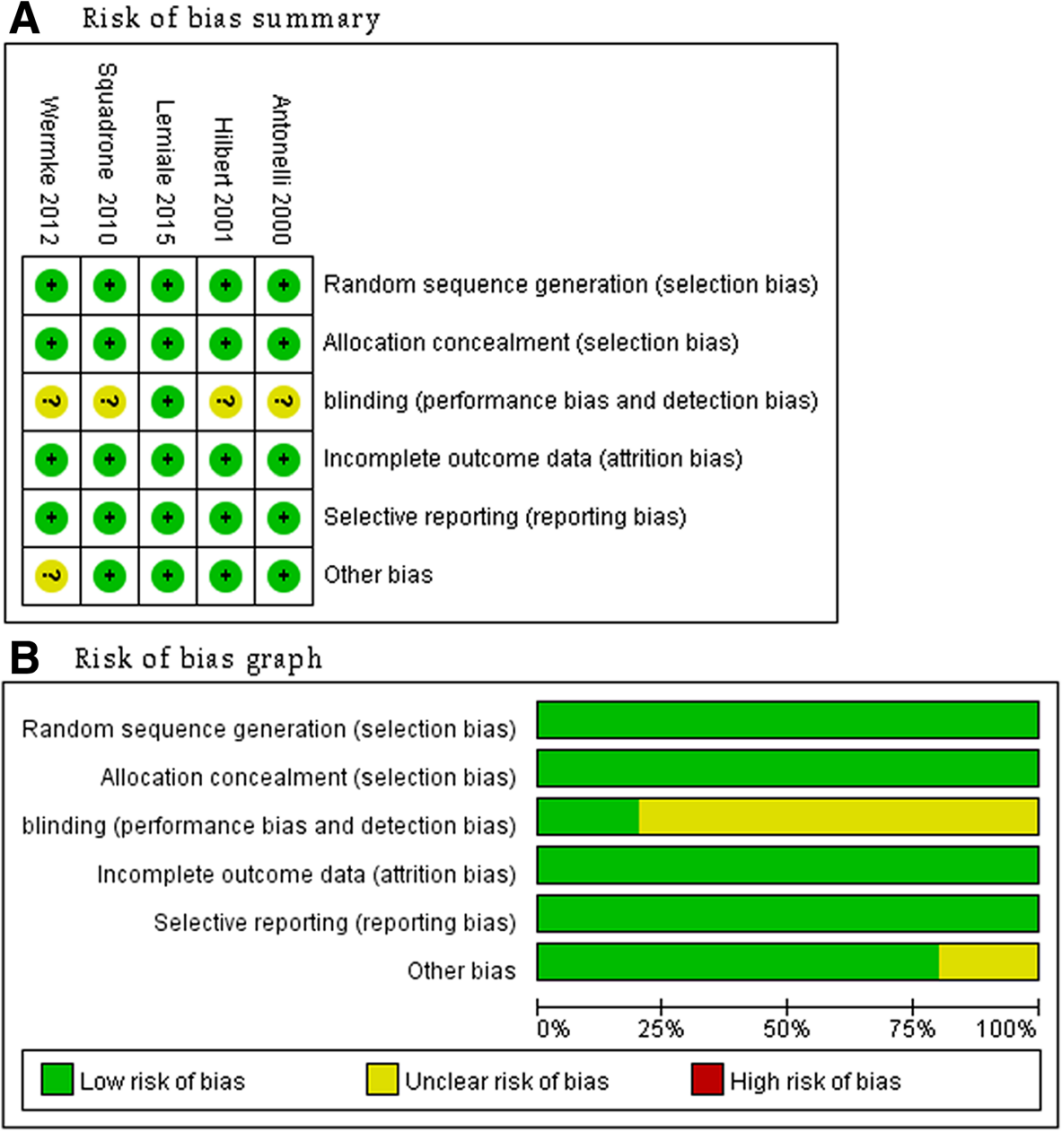
Risk-of-bias analysis

The main characteristics of the included RCTs are described in Table 1 and Additional file 1: Table S1, while the diagnostic criteria for ARF, treatment algorithms, and outcome data are described in Tables 2 and 3, and Additional file 1: Table S2. These studies were published between 2000 and 2015, with sample sizes ranging from 40 to 374 patients. Three studies [10, 11, 14] reported a moderate PaO_2_/FiO_2_ ratio (range 100–200) and two studies [12, 13] reported a mild PaO_2_/FiO_2_ ratio (range 200–300) in the enrolled patients. The immunocompromised patients in the five studies included patients with cancer (five trials) [10–14], patients with solid organ transplantation (two trials) [10, 14], patients participating in trials of other immunosuppressive agents (two trials) [11, 14], and patients with acquired immunodeficiency syndrome (one trial) [11]. Noninvasive pressure support ventilation (NIPSV) mode was used in four trials, with variable levels of positive end-expiratory pressure (PEEP) ranging from 2 to 10 cmH_2_O according to the target of oxygenation and patient tolerance [10, 11, 13, 14], whereas continuous positive airway pressure (CPAP) of 10 cmH_2_O was used in one trial [12]. The Cochrane risk of bias score varied across the studies (Fig. 2).

**Table 1:** Characteristics of the included studies

Study/year	Design	Country	Setting	Underlying conditions	Patient characteristics (NIV/Ctrl)					Primary outcome	Mortality follow up
					Patient number	Age, years	Disease severity	RR/min	PO_2_:FiO_2_		
Antonelli et al. 2000 [10]	P, RC, SC	Italy	ICU	Organ transplant	20/20	45/44	SAPS I 13 ± 4/13 ± 3	38 ± 3/37 ± 1	129/129	Need of intubation	28 days
Hilbert et al. 2001 [11]	P, RC, SC	France	ICU	Mixed immunocompromised	26/26	48/50	SAPS II 45 ± 10/42 ± 9	35 ± 3/36 ± 3	141/136	Need of intubation	28 days
Squadrone et al. 2010 [12]	P, RC, SC	Italy	Hematology ward	Hematologic malignancy	20/20	50/49	SAPS II 41.3 ± 6/42 ± 7	30/29	282/256	Need of intubation	90 days
Wermke et al. 2012 [13]	P, RC, SC	Germany	Hematology ward	Allogeneic HSCT	42/44	53/52	NR	NR	250–300	Mortality	5 years
Lemiale et al. 2015 [14]	P, RC, MC	France/Belgium	ICU	Mixed immunocompromised	191/183	64/61	SOFA 5 (3–7)/5 (3–7)	25 (21–30)/27 (21–31)	130/156	Mortality	180 days

*HFNC* heated and humidified high flow oxygen delivered by nasal cannula, *HSCT* hematopoietic stem cell transplantation, *ICU* intensive care unit, *MC* multi-center, *NIV* non-invasive ventilation, *NR* not reported, *P* prospective, *RC* randomized controlled, *RR* respiratory rate, *SAPS* simplified acute physiologic score, *SC* single-center, *SOFA* sequential organ failure assessment score

**Table 2:** Definition of criteria for acute hypoxemic respiratory failure and study treatment algorithm

Study	Criteria for acute hypoxemic respiratory failure	Study treatment algorithms
Antonelli et al. 2000 [10]	RR >35/min; PaO_2_/FiO_2_ < 200 while breathing oxygen; active contraction of accessory muscles of respiration or paradoxical abdominal motion	Ventilation algorithm: NIV via facemask; pressure support adjusted to obtain a Vt of 8–10 mL/kg, RR <25/min, the disappearance of accessory muscle activity and patient comfort. Control algorithm: patients received oxygen supplementation via a Venturi mask starting with an FiO_2_ ≥ 0.4, and adjusted to SpO_2_ > 90%
Hilbert et al. 2001 [11]	Pulmonary infiltrates and fever; severe dyspnea at rest; RR >30/min; PaO_2_/FiO_2_ < 200 while breathing oxygen	Ventilation algorithm: NIV via facemask; pressure support adjusted to obtain a Vt of 7–10 mL/kg; RR <25/min. PEEP was increased by 2 cmH_2_O, up to 10 cmH_2_O, adjusted to FiO_2_ ≤ 65% and SpO_2_ > 90%. Control algorithm: patients received oxygen through a Venturi mask. The rate of administration of oxygen was adjusted to SpO_2_ > 90%
Squadrone et al. 2010 [12]	Bilateral pulmonary infiltrates; SpO_2_ < 90% with room air; RR >25/min; respiratory symptom duration <48 h	Ventilation algorithm: CPAP via facemask or helmet at 10 cmH_2_O and FiO_2_ = 50%. Control algorithm: patients received oxygen through a Venturi mask
Wermke et al. 2012 [13]	RR >25/min; PaO_2_/FiO_2_ < 300 or SpO_2_ < 92% with room air	Ventilation algorithm: NIV via facemask; with pressure support of 15 cmH_2_O and an initial PEEP of 7 cmH_2_O; adjustments were according to capillary blood gas analysis and tolerance of patient. Control algorithm: patients received oxygen via nasal insufflation or full face mask initially set to 3 L/min. Adjustment of oxygen flow was left to physician’s discretion
Lemiale et al. 2015 [14]	PaO_2_ < 60 mmHg with room air; RR >30/min, or labored breathing or respiratory distress or dyspnea at rest; respiratory symptom duration <72 h	NIV algorithm: NIV via facemask; pressure support adjusted to obtain a Vt of 7–10 mL/kg ideal body weight; with an initial PEEP 2–10 cmH_2_O. The FiO_2_ and PEEP were adjusted to SpO_2_ ≥ 92%. Control algorithm: oxygenation modalities and the use of HFNC at clinician’s discretion

*PaO*_*2*_ */FiO*_*2*_ ratio of arterial pressure of oxygen/fraction of inspired oxygen, *SpO*_*2*_ pulse arterial oxygen saturation, *CPAP* continuous positive airway pressure, *HFNC* heated and humidified high flow oxygen delivered by nasal cannula, *ICU* intensive care unit, *PEEP* positive end expiratory pressure, *NIV* noninvasive ventilation, *RR* respiratory rate, *Vt* tidal volume

**Table 3:** Outcome of NIV and standard oxygen therapy for included studies

Study/year	ICU mortality (%)		Hospital mortality (%)		28-day mortality (%)		Long-term mortality (%)		Mortality in patients with ET (%)		Intubation rate (%)		Length of ICU stay (days)	
	NIV	Ctrl	NIV	Ctrl	NIV	Ctrl	NIV	Ctrl	NIV	Ctrl	NIV	Ctrl	NIV	Ctrl
Antonelli et al. 2000 [10]	20	50	35	55	NR	NR	NR	NR	100	71.4	20	70	7 ± 5	10 ± 6
Hilbert et al. 2001 [11]	38.5	69.2	50	80.8	NR	NR	NR	NR	100	100	46.2	76.9	7 ± 3	9 ± 4
Squadrone et al. 2010 [12]	NR	NR	15	75	NR	NR	NR	NR	100	100	10	70	0 (0–28)	28 (0–28)
Wermke et al. 2012 [13]	NR	NR	NR	NR	NR	NR	39	32	100	100	14.3	25	NR	NR
Lemiale et al. 2015 [14]	20.9	24.6	30.9	34.4	24.1	27.3.	39.6	45.3	NR	NR	38.2	44.8	7 (3–16)	6 (3–16)

*Ctrl* control, *ET* endotracheal intubation, *ICU* intensive care unit, *NIV* noninvasive ventilation, *NR* not reported

### Primary outcome

**Fig 3:**
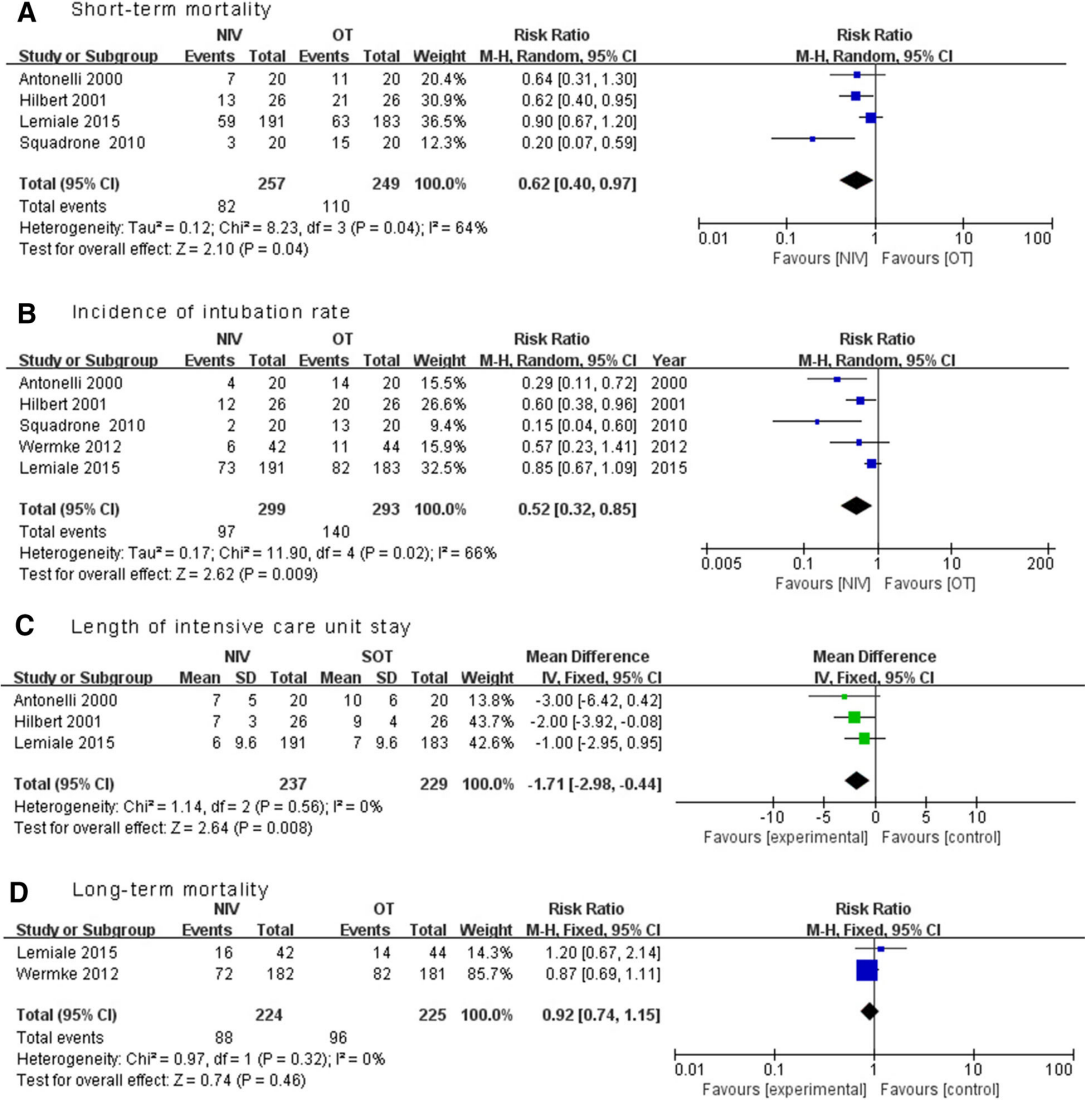
Effects of noninvasive ventilation (NIV) on immunocompromised patients. Forest plot showing the effect of NIV on short-term mortality (**a**), incidence of intubation rate (**b**), length of intensive care unit stay (**c**) and long-term mortality (**d**). *OT* oxygen therapy

Mortality was reported in all five RCTs. Short-term mortality data were available in four studies, [10–12, 14] In pooled analysis the use of NIV was associated with a significant reduction in short-term mortality (four trials; RR 0.62, 95% CI 0.40 to 0.97, *p* = 0.04), with significant heterogeneity among the studies (*I* ^2^ = 64%) (Fig. 3a). Therefore, we conducted sensitivity analyses to explore potential sources of heterogeneity. Exclusion of the trial by Squadrone and colleagues [12] significantly decreased the heterogeneity without altering the result (three trials; RR 0.76, 95% CI 0.59 to 1.00, *p* = 0.047; *I* ^2^ = 17%) [10, 11, 14]. In sensitivity analysis, both ICU and hospital mortality rates were significantly lower in the NIV group, while subgroup analysis confirmed a consistent reduction in mortality in patients with moderate hypoxemia, and patients with cancer or solid organ transplantation. However, use of NIV did not exhibit any beneficial effect on mortality in patients receiving immunocompromised agents or patients with mild hypoxemia (Table 4).

**Table 4:** Further analysis on mortality

	Studies number	Patient number	Event in NIV group	Event in control group	Risk ratio (95% CI)	*I* ^2^	*P*
Short-term mortality	4	506	82 of 257 (31.9%)	110 of 249 (44.2%)	0.73 (0.58, 0.91)	64%	0.04
ICU mortality	3	466	54 of 237 (22.8%)	73 of 229 (31.9%)	0.72 (0.53, 0.97)	33%	0.03
Hospital mortality	4	506	82 of 257 (31.9%)	110 of 249 (44.2%)	0.73 (0.58, 0.91)	64%	0.04
Patients with PaO_2_/FiO_2_ < 200	3	541	92 of 228 (40.4%)	114 of 227 (50.2%)	0.77 (0.61, 0.98)	13%	0.03
Patients with PaO_2_/FiO_2_ > 200	2	126	29 of 62 (46.8%)	45 of 64 (70.3%)	0.46 (0.09, 2.34)	88%	0.35
Patients with diagnosis of cancer and transplantation	5	507	85 of 258 (32.9%)	113 of 249 (45.4%)	0.68 (0.48, 0.97)	58%	0.03
Patients with diagnosis of drug-related immunosuppression	2	81	7 of 33 (21.2%)	5 of 30 (16.7%)	0.95 (0.48, 1.87)	0%	0.37

*ICU* intensive care unit, *NIV* noninvasive ventilation, *PaO*_*2*_ */FiO*_*2*_ ratio of arterial pressure of oxygen/fraction of inspired oxygen

### Secondary outcomes

Use of NIV was associated with significant reduction in the intubation rate (five trials, RR 0.52, 95% CI 0.31 to 0.87, *p* = 0.01; *I* ^2^ = 68%) (Fig. 3b) and length of ICU stay (three trials, MD −1.71 days, 95% CI −2.98 to −0.44, *p* = 0.008; *I* ^2^ = 0%)(Fig. 3c) [10, 11, 14], but not a decrease in long-term mortality (two trials; RR 0.92, 95% CI 0.74 to 1.15, *p* = 0.46) (Fig. 3d). [13, 14] There was significant heterogeneity in the outcome of intubation rate between the pooled RCTs. Further exclusion of any single RCT did not materially change the overall combined RR, which ranged from 0.42 (95% CI 0.24 to 0.74, *p* = 0.003) to 0.61 (95% CI 0.37 to 1.00, *p* = 0.05), while heterogeneity still existed (*I* ^2^ range 46–75%).

## Discussion

Our meta-analysis illustrated that early use of NIV could effectively reduce short-term mortality in immunocompromised patients with ARF when compared with oxygen therapy alone. In addition, the NIV strategy was associated with a reduction in the rate of endotracheal intubation and length of ICU stay.

Although our results are encouraging, several important issues merit detailed discussion. First, significant heterogeneity was observed between pooled studies in the primary outcome. This is not surprising, given the differences in the diagnostic criteria for ARF, treatment algorithms, and underlying diseases. Our sensitivity analyses showed that the trial by Squadrone and colleagues [12] probably contributed to the observed heterogeneity. Unlike other included trials, Squadrone and colleagues enrolled immunocompromised patients without a diagnosis of pneumonia, infection, or sepsis. Of note, these patients had a higher PaO_2_/FiO_2_ ratio, and were managed by CPAP rather than by NIPSV. After excluding this trial, the pooled result of the remaining studies still showed a reduction in mortality. Furthermore, we also demonstrated a significant reduction in the intubation rate and length of ICU stay in the NIV group, which added robustness to our primary outcome.

Second, our findings contradicted the results of the two latest RCTs [13, 14]. These two trials, although included in our meta-analysis, did not report a significant difference in clinical outcome (e.g. short-term or long-term mortality and intubation rate) among inpatients assigned to early NIV compared with oxygen therapy alone. Wermkeet al. [13] enrolled patients with mild hypoxemia, as suggested by a mean PO_2_/FiO_2_ ratio of 250 to 300. Moreover, 36.4% (16/44) of patients in the control group received NIV as a rescue therapy. The high crossover rate might have masked the beneficial effect, if any, of NIV in immunocompromised patients with ARF. This trial might also contribute to the negative findings in the subgroup of mild hypoxemia. In comparison, in the study of Lemiale and colleagues [14], a high-flow nasal cannula (HFNC) was used in both groups at the discretion of treating physicians.

Interestingly, HFNC was used more often in the oxygen group than in the NIV group (44% vs. 31%, *p* = 0.01). HFNC is a new technique that may deliver up to 100% humidified oxygen at a high flow rate. The advantages of HFNC include a high fraction of inspired oxygen to improve oxygenation, generation of flow-dependent PEEP (2–5 cmH_2_O) to improve alveolar recruitment, enhanced washout of nasopharyngeal dead space, and greater comfort in patients requiring oxygen therapy [17]. Several studies have shown that compared with conventional oxygen therapy, HFNC in immunocompetent patients with ARF could improve respiratory parameters, comfort and patient tolerance [18–20]. Moreover, in an observational cohort study of immunocompromised ICU patients with ARF, Coudroy and colleagues reported that use of HFNC was associated with a significant reduction in intubation rate and 28-day mortality compared to NIV (35% vs. 55%, *p* = 0.04, and 20% vs. 40%, *p* = 0.02, respectively) [21]. As a result, the use of HFNC could have greatly reduced the demand for IMV in control group, thereby diluting the benefits of NIV in these patients. More than this, it may also explain why there was no reduction in long-term mortality in the pooled analysis of patients in these two trials only. It is also noteworthy that in the study by Lemiale and colleagues [14], the overall mortality rate was much lower than in the other included RCTs (hospital mortality 32.6% vs. 45–65%). Thus, this study might provide a clue to the potential benefits of HFNC, or HFNC in combination with NIV, over NIV alone. Nevertheless, such a hypothesis should await validation by a large scale, well-designed RCT in the future.

Third, extremely high mortality rates were reported in immunocompromised patients who did not respond to conventional oxygen therapy or to NIV (Table 3); these rates were even higher than the mortality rate of 70–80% that is widely reported in previous studies [22, 23]. Despite the fact that the exact reasons remain to be clarified, such high mortality might support the recommendation against the use of IMV in this vulnerable patient population, thus favoring NIV as the first-line choice of therapy [1].

Finally, we also found that early use of NIV was associated with a significant reduction in the length of ICU stay. This encouraging result has added robustness to the conclusion that early NIV strategy is effective in immunocompromised patients with ARF. Although in clinical practice, ICU discharge is not always determined by the patient condition and needs to be individualized [24]; the less likely that a patient requires tracheal intubation, the more likely that physicians feel comfortable about the patient being discharged from ICU.

The current meta-analysis has provided evidence to support and expand the weak suggestion in the 2011 Canadian guidelines [1], i.e. use of noninvasive positive-pressure ventilation (NPPV) in immunocompromised patients with ARF. However, this study has some limitations. First, only five RCTs were included in the current meta-analysis, while four of them had a sample size of 40 to 86 patients, which more likely resulted in overestimation of the treatment effect than in studies with larger sample sizes. Second, significant heterogeneity was observed in some of our outcomes. There were remarkable differences among the included trials in the adopted definition of ARF, timing and duration of oxygen therapy or NIV, and indications for endotracheal intubation, which might lead to the observed heterogeneity, and further impair the robustness of our findings. Third, the uneven distribution of different underlying diseases among the included studies might also exert a prognostic value [4, 25, 26]. Although predefined subgroup analyses had been performed, the results should be interpreted with caution due to the small number of patients in some disease categories, i.e. patients receiving immunocompromised drugs or patients with acquired immunodeficiency syndrome.

## Conclusions

In summary, based on the available data, our results demonstrate that compared with oxygen therapy, early respiratory support with NIV would significantly reduce mortality, intubation rate and length of ICU in immunocompromised patients with ARF of various origins. Large-scale, well-designed RCTs will be needed to define the subgroup of patients that are most likely to benefit from this strategy.

## Key messages

- Early use of NIV significantly reduced overall mortality, intubation rate and length of ICU stay in immunocompromised patients with ARF of various origins, when compared with administration of oxygen therapy.
- Further larger adequately powered RCTs are warranted to identify in which selected patients NIV could be more beneficial before the wider application of this ventilator strategy.

## Additional file

Additional file 1: Table S1. Characteristics of patients enrolled in the included studies, categorized by type of immunosuppression and cause for acute respiratory failure. **Table S2.** Summary of mortality and intubation rates in patients categorized by type of immunosuppression and cause of acute respiratory failure. (DOCX 31 kb)

## Abbreviations

ARF: acute respiratory failure
CI: confidence interval
CPAP: continuous positive airway pressure
HFNC: high-flow nasal cannula
ICU: intensive care unit
IMV: invasive mechanical ventilation
IQR: interquartile range
MD: mean differences
NIMV: noninvasive mechanical ventilation
NIPSV: noninvasive pressure support ventilation
NIV: noninvasive ventilation
NPPV: noninvasive positive-pressure ventilation
PaO_2_/FiO_2_: ratio of arterial pressure of oxygen/fraction of inspired oxygen
PEEP: positive end-expiratory pressure
RCT: randomized controlled trial
RR: risk ratio
SD: standard deviation
