# Supplementary material for: Use of noninvasive ventilation in immunocompromised patients with acute respiratory failure: a systematic review and meta-analysis
Source: Crit Care. 2017 Jan 7;21:4. doi: 10.1186/s13054-016-1586-9 (PMC5219799; doi:10.1186/s13054-016-1586-9)
Supplement: Additional file 1: Table S1. — Characteristics of patients enrolled in the included studies, categorized by type of immunosuppression and cause for acute respiratory failure. Table S2. Summary of mortality and intubation rates in patients categorized by type of immunosuppression and cause of acute respiratory failure. (DOCX 31 kb) [file 13054_2016_1586_MOESM1_ESM.docx]

**Supplementary materials for “Use of noninvasive ventilation in immunocompromised patients with acute respiratory failure: a systematic review and meta-analysis”**

Hui-Bin Huang, Biao Xu, Guang-Yun Liu, Jian-Dong Lin, Bin Du

**Contents**

**Table S1**.Characteristics of patients enrolled in included studies, categorized by type of immunosuppression and cause for acute respiratory failure

**Table S2**. Summary of mortality and intubation rates in patients categorized by type of immunosuppression and cause for acute respiratory failure

**Correspondence:** Bin Du

Medical ICU, Peking Union Medical College Hospital, Peking Union Medical College and Chinese Academy of Medical Sciences,

1 Shuai Fu Yuan, Beijing, 100730, PR China, Tel: 8610-6915-5036 Fax: 8610-6915-4036, Email: dubin98@gmail.com

**Table S1. Characteristics of patients enrolled in included studies, categorized by type of immunosuppression and cause for acute respiratory failure**

|  | Antonelli et al. ^[10]^ | |  | Hilbert et al.^[11]^ | |  | Squadrone et al. ^[12]^ | |  | Wermke et al.^[13]^ | |  | Lemiale et al.^[14]^ | |
| --- | --- | --- | --- | --- | --- | --- | --- | --- | --- | --- | --- | --- | --- | --- |
|  | **NIV** | **Ctrl** |  | **NIV** | **Ctrl** |  | **NIV** | **Ctrl** |  | **NIV** | **Ctrl** |  | **NIV** | **Ctrl** |
| Number of patients | 20 | 20 |  | 26 | 26 |  | 20 | 20 |  | 42 | 44 |  | 191 | 183 |
| Included patients with infection? | Yes | |  | Yes | |  | No | |  | Yes | |  | Yes | |
| Type of immunosuppression |  |  |  |  |  |  |  |  |  |  |  |  |  |  |
| Cancer |  |  |  |  |  |  |  |  |  |  |  |  | 162 | 155 |
| Hematologic malignancies |  |  |  | 15 | 15 |  | 20 | 20 |  | 42 | 44 |  | 125 | 113 |
| Solid tumors | 20 | 20 |  |  |  |  |  |  |  |  |  |  | 37 | 42 |
| Immunosuppressive drugs |  |  |  | 9 | 9 |  |  |  |  |  |  |  | 29 | 28 |
| Non-transplant-related reasons |  |  |  | 6 | 5 |  |  |  |  |  |  |  | 16 | 17 |
| After solid organ transplantation |  |  |  | 3 | 4 |  |  |  |  |  |  |  | 13 | 11 |
| HIV |  |  |  | 2 | 2 |  |  |  |  |  |  |  |  |  |
| Cause for ARF |  |  |  |  |  |  |  |  |  |  |  |  |  |  |
| Pneumonia† | 7 | 4 |  |  |  |  |  |  |  |  |  |  | 134 | 123 |
| Bacterial pneumonia |  |  |  |  |  |  |  |  |  |  |  |  | 87 | 83 |
| Pneumocystis jirovecii pneumonia |  |  |  |  |  |  |  |  |  |  |  |  | 22 | 21 |
| Viral pneumonia |  |  |  |  |  |  |  |  |  |  |  |  | 19 | 15 |
| Invasive pulmonary aspergillosis |  |  |  |  |  |  |  |  |  |  |  |  | 6 | 4 |
| Lung involvement by underlying disease |  |  |  |  |  |  |  |  |  |  |  |  | 21 | 15 |
| Drug-related pulmonary toxicity |  |  |  |  |  |  |  |  |  |  |  |  | 10 | 9 |
| Cardiogenic pulmonary edema | 4 | 5 |  |  |  |  |  |  |  |  |  |  | 7 | 2 |
| ARDS (extrapulmonary etiology) | 3 | 5 |  |  |  |  |  |  |  |  |  |  | 11 | 12 |
| Diffuse intra-alveolar hemorrhage |  |  |  |  |  |  |  |  |  |  |  |  | 0 | 2 |
| Mucous plugging or atelectasis | 5 | 5 |  |  |  |  |  |  |  |  |  |  |  |  |
| Other | 1 | 1 |  |  |  |  |  |  |  |  |  |  | 8† | 20† |

ARDS, acute respiratory distress syndrome; Ctrl, control; NIV, non-invasive ventilation; †: included patients with other identified causes (n = 11) and patients with no identified causes (n = 17),

of the other identified causes: Large pleural effusions (n = 4), pulmonary infarction revealing pulmonary embolism (n = 5), disseminated toxoplasmosis (n = 1), and pain-related atelectasis (n = 1);

**Table S2. Summary of mortality and intubation rates in patients categorized by type of immunosuppression and cause for acute respiratory failure**

|  | Antonelli et al. ^[10]^ | |  | Hilbert et al.^[11]^ | |  | Squadrone et al. ^[12]^ | |  | Wermke et al.^[13]^ | |  | Lemiale et al.^[14]^ | |
| --- | --- | --- | --- | --- | --- | --- | --- | --- | --- | --- | --- | --- | --- | --- |
|  | **NIV** | **Ctrl** |  | **NIV** | **Ctrl** |  | **NIV** | **Ctrl** |  | **NIV** | **Ctrl** |  | **NIV** | **Ctrl** |
| Mortality rate (n/N) |  |  |  |  |  |  |  |  |  |  |  |  |  |  |
| Types of immunosuppression |  |  |  |  |  |  |  |  |  |  |  |  |  |  |
| Cancer | 4/20 | 10/20 |  | 8/15 | 14/14 |  | 3/20 | 15/20 |  | 16/42 | 14/44 |  | 43/162 | 41/155 |
| Immunosuppressive drugs |  |  |  | 4/9 | 6/9 |  |  |  |  |  |  |  | 7/33 | 5/30 |
| HIV |  |  |  | 1/2 | 1/2 |  |  |  |  |  |  |  |  |  |
| Cause for ARF |  |  |  |  |  |  |  |  |  |  |  |  |  |  |
| Pneumonia† | 1/7 | 1/4 |  |  |  |  |  |  |  |  |  |  |  |  |
| Lung involvement by underlying disease |  |  |  |  |  |  |  |  |  |  |  |  |  |  |
| Drug-related pulmonary toxicity |  |  |  |  |  |  |  |  |  |  |  |  |  |  |
| Cardiogenic pulmonary edema | 0/4 | 4/5 |  |  |  |  |  |  |  |  |  |  |  |  |
| ARDS (extrapulmonary etiology) | 3/3 | 4/5 |  |  |  |  |  |  |  |  |  |  |  |  |
| Diffuse intra-alveolar hemorrhage |  |  |  |  |  |  |  |  |  |  |  |  |  |  |
| Mucous plugging or atelectasis | 0/5 | 1/5 |  |  |  |  |  |  |  |  |  |  |  |  |
| Other | 0/1 | 0/1 |  |  |  |  |  |  |  |  |  |  |  |  |
|  |  |  |  |  |  |  |  |  |  |  |  |  |  |  |
| Intubation rate (n/N) |  |  |  |  |  |  |  |  |  |  |  |  |  |  |
| Types of immunosuppression |  |  |  |  |  |  |  |  |  |  |  |  |  |  |
| Cancer | 4/20 | 14/20 |  | 8/15 | 14/14 |  | 2/20 | 14/20 |  | 6/42 | 11/44 |  |  |  |
| Immunosuppressive drugs |  |  |  | 3/9 | 5/9 |  |  |  |  |  |  |  |  |  |
| HIV |  |  |  | 1/2 | 1/2 |  |  |  |  |  |  |  |  |  |
| Cause for ARF |  |  |  |  |  |  |  |  |  |  |  |  |  |  |
| Pneumonia† | 3/7 | 3/4 |  |  |  |  |  |  |  |  |  |  |  |  |
| Lung involvement by underlying disease |  |  |  |  |  |  |  |  |  |  |  |  |  |  |
| Drug-related pulmonary toxicity |  |  |  |  |  |  |  |  |  |  |  |  |  |  |
| Cardiogenic pulmonary edema | 0/4 | 5/5 |  |  |  |  |  |  |  |  |  |  |  |  |
| ARDS (extrapulmonary etiology) | 1/3 | 4/5 |  |  |  |  |  |  |  |  |  |  |  |  |
| Diffuse intra-alveolar hemorrhage |  |  |  |  |  |  |  |  |  |  |  |  |  |  |
| Mucous plugging or atelectasis | 0/5 | 2/5 |  |  |  |  |  |  |  |  |  |  |  |  |
| Other | 0/1 | 0/1 |  |  |  |  |  |  |  |  |  |  |  |  |

ARDS, acute respiratory distress syndrome; Ctrl, control; NIV, non-invasive ventilation;
